# Prevalence and Risk Factors Associated With Inflammatory Bowel Disease in Patients Using Proton-Pump Inhibitors: A Population-Based Study

**DOI:** 10.7759/cureus.34088

**Published:** 2023-01-23

**Authors:** Somtochukwu Onwuzo, Antoine Boustany, Hadi Khaled Abou Zeid, Asif Hitawala, Ashraf Almomani, Chidera Onwuzo, Favour Lawrence, Jessy Mascarenhas Monteiro, Chidera Ndubueze, Imad Asaad

**Affiliations:** 1 Internal Medicine, Cleveland Clinic Foundation, Cleveland, USA; 2 Internal Medicine, University of Balamand, Koura, LBN; 3 Internal Medicine, General Hospital Lagos Island, Lagos, NGA; 4 Internal Medicine, Ross University School of Medicine, Bridgetown, BRB; 5 Internal Medicine, University of Port Harcourt Teaching Hospital, Port Harcourt, NGA

**Keywords:** database, population-based study, inflammatory bowel disease, crohn’s disease, ulcerative colitis, proton pump inhibitor

## Abstract

Background and aim

Proton pump inhibitor (PPI) is a heavily prescribed medication in the United States that is used to treat several gastrointestinal disorders. Although it has been considered to be safe compared to other medications, multiple gastrointestinal side effects have been reported. These effects of PPIs might stem from the progressive alteration of the intestinal microbiome. Patients with inflammatory bowel disease (IBD) using PPI are also seen to be less likely to achieve remission. However, in the current literature, there is very little evidence of the risk of developing IBD in patients who have been using PPIs. Therefore, our aim was to perform a cross-sectional population-based study with in-depth analysis to assess the prevalence and risk factors of IBD amongst PPI users in the United States.

Methodology

A validated multicenter and research platform database of more than 360 hospitals from 26 different healthcare systems across the United States was utilized to construct this study. A cohort of patients with a diagnosis of ulcerative colitis (UC) and Crohn’s disease (CD) between 1999-2022 was identified using the Systematized Nomenclature of Medicine-Clinical Terms (SNOMED-CT). Patients aged 18 to 65 years were included. We excluded any individual who had a diagnosis of chronic liver disease, autoimmune disease (excluding IBD), or cancer. The risk of IBD was calculated using a multivariate regression analysis to account for potential confounders including non-steroidal anti-inflammatory drugs (NSAIDs) use, smoking, patients who have had a diagnosis of alcoholism, gastroesophageal reflux disease (GERD), irritable bowel syndrome (IBS), and metabolic syndrome. A two-sided P-value <0.05 was considered statistically significant, and all statistical analyses were performed using R version 4.0.2 (R Foundation for Statistical Computing, Vienna, Austria, 2008).

Results

A total of 79,984,328 individuals were screened in the database and 45,586,150 patients were selected in the final analysis after accounting for inclusion and exclusion criteria. Using multivariate regression analysis, the risk of developing UC and CD was calculated. The odds of having UC amongst patients on PPI was 2.02 (95%CI 1.98-2.06), P-value <0.001. Similarly, the odds of having CD were high amongst PPI users (OR 2.79, 95%CI 2.75-2.84), P- value <0.001

Conclusion

Our study demonstrates that patients on PPIs are frequently found to have UC and CD even when adjusting for common risk factors. Hence, we urge clinicians to be aware of this association in order to limit unnecessary prescriptions of PPIs, especially for patients who are at risk for autoimmune diseases.

## Introduction

Proton pump inhibitors (PPIs) are commonly prescribed medications widely used for acid suppression in different gastrointestinal diseases [1-5]. In fact, PPIs constitute the first-line therapy for gastroesophageal reflux disease (GERD), esophagitis, *Helicobacter pylori* infection, Zollinger-Ellison Disease, and peptic ulcer disease (PUD) [1-7]. Ever since its introduction to the United States (US) market, the number of PPI users has been increasing annually [3,5,6,8]. In 2016 alone, more than 150 million PPIs were prescribed in the US, not counting over-the-counter (OTC) medications [9]. Despite their relatively safe use, PPIs have been recently associated with several side effects, especially with long-term use [3,6,8,10,11]. Several studies found an increased risk of osteoporosis, hypomagnesemia, and gastrointestinal and respiratory infections linked to PPI intake [3,4,6,7]. Significant drug-drug interactions and cardiovascular and renal problems were also noted in patients using PPIs [3,6,7]. Besides this, a meta-analysis of randomized clinical trials demonstrated a decreased likelihood of inflammatory bowel disease (IBD) remission on Infliximab in PPI users [12], and Schwartz et al. found an increased risk of IBD in children who were prescribed PPIs [8]. The potential relationship between IBD and PPIs use has been described in a few other articles in the literature [12,13]. Nevertheless, there is still little evidence assessing the risk of developing IBD in PPI users. Hence, we aim in this article to perform a population-based study with in-depth analysis of the risk of developing IBD in PPIs users

## Materials and methods

Our cohort’s data were obtained using a validated, multicentered, and daily-updated database, IBM® Explorys platform (IBM Corp., Armonk, New York, US). Explorys has electronic health records of 26 different healthcare systems with a total of about 360 hospitals and more than 70 million patients across the US. It utilizes Systematized Nomenclature of Medicine-Clinical Terms (SNOMED-CT) for the definition of the diseases and pools large outpatient as well as inpatient deidentified data that can be formulated into numerous cohorts according to the clinical element being studied. Explorys does not record individual patient data such as laboratory or imaging results. The approval of an Institutional Review Board is not required since Explorys is a Health Insurance Portability and Accountability Act (HIPAA)-compliant platform. The use of this database has been validated in multiple fields including cardiology, hematology, and gastroenterology.

Patient selection 

A cohort of patients with a SNOMED-CT diagnosis of ulcerative colitis (UC) and Crohn’s disease (CD) between 1999 and May 2022 was identified. Patients aged 18 to 65 years were included. We excluded any individual who has had a diagnosis of chronic liver disease, autoimmune disease (excluding IBD), or cancer.

Covariates

Confounding factors associated with UC and CD were identified and collected if SNOMED-CT diagnoses were available. These were non-steroidal anti-inflammatory drug (NSAID) use, smoking, alcoholism, GERD, irritable bowel syndrome (IBS), and metabolic syndrome.

Statistical analysis 

Prevalence of PPI use, UC, and CD were calculated by dividing the respective number of subjects by the total number of subjects in the Explorys database. To account for confounding from the covariates listed above, we conducted 1024 searches to explore every probability, with PPI as one of the variables. A univariate analysis was conducted initially for all the variables, followed by multivariate analysis. Statistical analysis was performed R version 4.0.2 (R Foundation for Statistical Computing, Vienna, Austria, 2008) and for all analyses, a two-sided p-value of <0.05 was considered statistically significant. Multivariate analysis was performed to adjust for multiple factors including the use of NSAIDs, smoking, alcoholism, GERD, IBS, and metabolic syndrome.

## Results

A total of 79,984,328 individuals were screened in the database and 45,586,150 patients were selected in the final analysis after accounting for inclusion and exclusion criteria. There was a total of 95,440 patients with UC with a prevalence rate of 210 per 100,000 and 131,010 patients with CD with a prevalence rate of 288 per 100,000 (Table 1). There was an increased prevalence of UC and CD among NSAID users and subjects with GERD.

**Table 1:** Baseline characteristics of patients with ulcerative colitis, Crohn’s disease, and controls GERD: gastroesophageal reflux disease; NSAID: non-steroidal anti-inflammatory drug; PPI: proton pump inhibitor

	Ulcerative colitis (%)	Crohn’s disease (%)	Control (%)
Metabolic syndrome	510 (0.53)	700 (0.53)	114,200 (0.25)
Irritable bowel syndrome	9,120 (9.55)	12,420 (9.48)	480,180 (1.05)
GERD	20,880 (21.87)	29,270 (22.34)	2,692,630 (5.93)
Alcohol Abuse	2,620 (2.74)	3,650 (2.78)	687,130 (1.51)
NSAID	66,210 (69.37)	83,660 (63.85)	13,253,700 (29.21)
Smokers	10040 (10.52)	16,460 (12.56)	2,368,010 (5.22)
PPI	30080 (31.51)	44,210 (33.75)	3,358,830 (7.40)
Total	95,440	131,010	45,359,700

In univariate analysis, the odds of having UC amongst patients on PPIs was 5.02 (95%CI 4.94-5.10). It was also high in patients with IBS (OR 8.58, 95%CI 8.36-8.80), GERD (OR 4.11, 95%CI 4.04-4.18), and NSAIDs use (OR 4.88, 95%CI 4.81-4.95) (Table 2). On the other hand, the odds of having CD were high amongst PPI users (OR 5.93, 95% CI 5.86-6.01). The odds also remained high for subjects with IBS (OR 8.93, 95%CI 8.74-9.11), GERD (OR 4.35, 95%CI 4.29-4.41), NSAID use (OR 3.83, 95%CI 3.79-3.88), and smokers (OR 2.56, 95% CI 2.52-2.61) (Table 2).

**Table 2:** The risk of developing ulcerative colitis and Crohn’s disease using univariate analysis IBS: irritable bowel syndrome; GERD: gastroesophageal reflux disease; NSAID: non-steroidal anti-inflammatory drug; PPI: proton pump inhibitor

	Ulcerative colitis OR (95% CI)	p-value	Crohn’s disease OR (95% CI)	p-value
Metabolism syndrome	2.20 (2.00-2.42)	<0.001	2.22 (2.11-2.46)	<0.001
IBS	8.58 (8.36-8.80)	<0.001	8.93 (8.74-9.11)	<0.001
GERD	4.11 (4.04-4.18)	<0.001	4.35 (4.29-4.41)	<0.001
Alcoholism	1.80 (1.73-1.88)	<0.001	1.85 (1.79-1.92)	<0.001
NSAID	4.88 (4.81-4.95)	<0.001	3.83 (3.79-3.88)	<0.001
Smoking	1.98 (1.93-2.02)	<0.001	2.56 (2.52-2.61)	<0.001
PPI	5.02 (4.94-5.10)	<0.001	5.93 (5.86-6.01)	<0.001

In multivariate analysis, the odds of having UC amongst patients on PPI was 2.02 (95%CI 1.98-2.06). The odds remained high in patients with IBS (OR 3.63, 95%CI 3.53-3.73), GERD (OR 1.40, 95%CI 1.37-1.44), and NSAID use (OR 3.67, 95%CI 3.62-3.74). Similarly, the odds of having CD were high amongst PPI users (OR 2.79, 95%CI 2.75-2.84), IBS (OR 3.65, 95%CI 3.57-3.73), GERD (OR 1.32, 95%CI 1.29-1.34), NSAID use (OR 2.49, 95% CI 2.46-2.52), and smokers 1.22 (95%CI 1.20-1.24). These results demonstrate that patients on PPIs are frequently found to have UC and CD even when adjusting for common risk factors. Smoking, metabolic syndrome, and alcoholism were found to be protective factors to the development of UC with OR 0.90 (95% CI 0.87-0.92), OR 0.90 (95%CI 0.82-0.99), and OR 0.95 (95%CI 0.91-0.99), respectively (Table 3).

**Table 3:** Risk of developing ulcerative colitis and Crohn’s disease using stepwise multivariate regression analysis IBS: irritable bowel syndrome; GERD: gastroesophageal reflux disease; NSAID: non-steroidal anti-inflammatory drug; PPI: proton pump inhibitor

	Ulcerative colitis OR (95% CI)	P-value	Crohn’s disease OR (95% CI)	P-value
Metabolic syndrome	0.90 (0.82-0.99)	0.04	0.93 (0.86-1.00)	0.07
IBS	3.63 (3.53-3.73)	<0.001	3.65 (3.57-3.73)	<0.001
GERD	1.40 (1.37-1.44)	<0.001	1.32 (1.29-1.34)	<0.001
Alcoholism	0.95 (0.91-0.99)	0.04	0.91 (0.88-0.94)	<0.001
NSAID	3.67 (3.62-3.74)	<0.001	2.49 (2.46-2.52)	<0.001
Smoking	0.90 (0.87-0.92)	<0.001	1.22 (1.20-1.24)	<0.001
PPI	2.02 (1.98-2.06)	<0.001	2.79 (2.75-2.84)	<0.001

## Discussion

Our study, which is the largest population-based study in the US, showed 2.02 and 2.79 increased odds of developing UC and CD, respectively, in patients who have been using PPI after adjusting for potential confounding variables including metabolic syndrome, irritable bowel syndrome, GERD, alcohol abuse, NSAIDs, and smoking.

PPIs work by inhibiting the proton-potassium pump (H+/K+ ATPase) found on parietal cells, and therefore block acid secretion into the stomach for 24 hours [1,2,7,10]. PPIs are metabolized by the CYP2C19 enzyme into their active metabolite [2,7]. Although generally safe to use, many adverse events and side effects have been linked to PPI intake. PPIs were proven to cause vitamin deficiency, electrolyte disturbances, and increased risk of fracture due to the decreased intestinal absorption of necessary vitamins and elements secondary to decreased gastric acidity [2,6,7]. Moreover, patients on PPI were more prone to develop gastrointestinal and pulmonary infections. This observation was thought to be due to the changes in the gastrointestinal and respiratory microbiota caused by PPI use [2]. Additionally, it was found that PPIs resulted in adverse cardiovascular events through different mechanisms [2,7]. To elaborate, PPIs decreased the efficiency of medications used in the treatment of heart diseases by irreversibly binding to the CYP450 enzyme [2,6,7]. Similarly, PPIs promoted hypomagnesemia, endothelial dysfunction, and platelet stimulation, which pose a risk for cardiovascular problems [2]. 

IBD is a chronic inflammation of the gastrointestinal tract attributed to dysfunction in the genes, immunity, environment, and microbiota [11,14]. Medications that disrupt the gut microbiota like antibiotics are considered major risk factors for developing IBD [11]. Surprisingly, a limited number of papers discussed the relationship between IBD and PPIs use. One study found that PPI users are at an increased risk of IBD-related hospitalizations, surgical procedures, and UC flare-ups [12]. In their meta-analysis, Lu et al. demonstrated decreased remission and increased hospitalization rates in IBD patients on Infliximab who used PPIs concomitantly. However, when comparing CD and UC remissions in PPI users, their data showed a significant decrease in remission rate in CD compared to a nonsignificant change in UC remission rate, but was not significant for UC development. These results were attributed to immunity dysfunction and gut dysbiosis [14]. Similarly, two meta-analyses proved a positive relationship between PPI use and the risk of IBD development [11,14]. A case-control study conducted on children showed that PPI users were 3.6 times more likely to develop IBD [13].

This population-based study comes in line with previously published articles and helps solidify the evidence behind the positive relationship between PPI use and IBD development. Nonetheless, the pathophysiology behind this correlation is still unclear [14]. One of the hypotheses claims that IBD results from the inflammatory state precipitated by PPI-induced electrolyte disturbances [14]. Another hypothesis is that PPIs cause dysfunction in tight junctions by modifying the gastric epithelium cytoskeleton [11,14]. The most plausible hypothesis states that PPIs provoke a change in the gut microbiota that triggers an inflammation and immune response responsible for the development of IBD [11-14]. 

In our study, smoking was a protective factor in the development of UC. This finding is similar to the results from a few studies. In a meta-analysis by Mahid et al. examining the relationship between smoking and IBD, results show current smoking had a protective effect on the development of UC when compared with controls (OR 0.58, 95%CI 0.45-0.75) [15]. Also, similar findings were also noted by Boyko et al. in a case-control study where smoking histories before the date of the onset of UC were compared in cases and an equal number of controls matched for age and sex; the results showed that the relative risk of UC among current cigarette smokers as compared with nonsmokers was 0.6 (95%CI 0.4-1.0); however, among former cigarette smokers, it was 2.0 (95%CI 1.1-3.7). These values remained after adjustment for socioeconomic factors and for coffee and alcohol consumption [16]. Interestingly, our study also shows that alcoholism might be associated with decreased risk of UC; these results are in line with a study by Boyko et al. where results from a population-based, case-control study assessing the risk of UC associated with coffee and alcohol use showed that moderate alcohol consumption may lower UC incidence [17]

Our study has several strengths. Being a multicenter study with a large sample size derived from the US population, comprising various ages and ethnicities make our results reliable and generalizable. Additionally, data utilized in this study was collected for a very long period of time and was also controlled for important variables that play a role in the pathogenesis of IBD. Our study showed that these factors were independently associated with increased prevalence of UC and CD, which have been well documented in the literature.

Limits of the study

Limitation to our study includes its retrospective nature and inability to establish causality. Being a database study, there is always a concern regarding selection bias. Furthermore, given that this database is HIPAA-compliant and anonymous, it is not possible to verify the accuracy of the diagnoses made due to the lack of information regarding the diagnostic tests, diagnostic imaging, diagnostic criteria, and duration of medication intake. Hence, further in-depth analysis is not feasible. Though power is one of the strengths of large database studies like this, there is always a possibility of over-estimation of odd ratios.

## Conclusions

Our study demonstrates that PPI users are frequently found to have UC and CD. While disruption in gut microbiota has been the proposed link, the association between IBD and PPI use still needs further exploration on whether longterm use of PPIs in treatment of other gastrointestinal disorders leads to increased risk for IBD or reflex initiation of PPIs during hospitalization from gastrointestinal bleeding in the setting of IBD flare without prompt discontinuation on hospital discharge explains the association. Nevertheless, we urge clinicians to be aware of this association in order to limit unnecessary prescription of PPIs, especially in patients who are at risk for autoimmune diseases. 
